# Medical Opinion and Movement

**Published:** 1909-06-19

**Authors:** 


### Medical Opinion and Movement.

THE discovery of the Specific Organism of Syphilis has led to considerable research in regard to the relation between this disease and still-birth. Dr. Gräfenburg, examining 50 macerated stillborn fœtuses, found the treponemes in 39, that is in 78 per cent., and he points out that this may be taken as a minimum, as a negative result is not conclusive against the presence of the disease. In 44 confinements of syphilitic patients, maceration of the fœtus occurred 38 times, that is a proportion of 84 per cent. So that one may conclude that maceration of the fœtus is a strong indication of the presence of syphilis. In only three cases the infants went to full term, but the author has never found the treponemes in the fœtus before the thirteenth week. Colles' law appears to hold good in the light of these researches. Only a few of the mothers showed any sign of the disease, and on the other hand in two cases of pronounced syphilitic lesions in the mother examination of the infants failed to discover the specific organism. The treponemes are seldom found in the placenta, but in the cord, in the walls of the bloodvessels close to the umbilicus. The author gives details for the search for treponemes at this site, and considers that confirmation of their presence in this way may be of considerable value in cases where the diagnosis is doubtful.

AT a meeting of the Académie des Sciences Monsieur Devaux reports some interesting observations upon the relation between Sleep and the Retention of Fluid in the Tissues. He suggests that every organ in a state of active function increases its osmotic power and that the œdema and thirst of fatigue are due to this osmotic attraction of fluid to the tissue in activity. Histological changes in experimental insomnia point to a relation between fatigue and the need of sleep and retention of fluid in the tissues. In further support of this idea the author recalls the swelling of the eyelids and face in consequence of a heavy and prolonged sleep. He has put the question to experimental test in the following manner: A light weight is placed upon the forearm, and the duration of the impression is noted and compared when the subject is awake and asleep. With 25 subjects experimented upon in this way the mean persistence of the impression during sleep was 4 minutes 18 seconds, and in the waking state 2 minutes 38 seconds. From these observations Monsieur Devaux concludes that the œdema of sleep is not confined to the face, but is a general œdema of the tissues, and that even light sleep is accompanied by an interstitial retention of fluid of the whole organism, and he thinks that this would especially apply to the brain.

SUCCESSFUL as the operation of Gastro-enterostomy has proved to be in a number of morbid conditions of the stomach, digestive disturbances are not an infrequent consequence of this form of surgical interference. Dr. S. Jonas has endeavoured to discover the cause for these troubles by means of radioscopic examination after a test meal with bismuth. He finds that in cases in which the operation is completely successful, and there are no disturbances of digestion, the food leaves the stomach by the orifice of the gastro-enterostomy immediately, and that the stomach does not retain the food at all. On the other hand, when the patient experiences pain some time after a meal, the food remains for a variable length of time in the stomach. This may be due to a narrowing of the intestinal orifice or to the position of the orifice in the wall of the stomach. On entering the stomach the food naturally falls to the lowest part of the greater curvature, and if the orifice is there it can pass out at once, but according to the author the intestinal orifice is very often higher up, and the food is only evacuated by a change in position of the patient, or after excessive peristalsis of the stomach wall, which gives rise to pain. Moreover, this stasis of the food causes a local dilatation which increases the trouble. The practical moral, therefore, of this examination is to make the orifice of the gastro-enterostomy as low as possible in the stomach wall. In cases in which this is impossible the author thinks the tendency to dilatation and the formation of a *cul de sac* may be controlled by an abdominal belt and by massage, and he also suggests that by means of the *x*-rays the position of the body might be ascertained in which the stomach contents are most easily evacuated through the intestinal orifice.

APROPOS of the use of Bismuth Salts for the Radioscopic Examination of the Digestive Tract, several cases of bismuth poisoning have been reported recently as a consequence of their administration in large quantities for this purpose. The chief features of the condition are a severe stomatitis with the formation of a false membrane on the lips, tonsils, etc., salivation, nausea, dysphagia, vomiting, meteorism, and diarrhœa. There is also a blackish coloration of the tissues, especially of the tongue, lips, tonsils, gums, and the rest of the alimentary tract. Rarely this coloration extends to the skin of the trunk and limbs. There is diminution of urine excreted, and there may be nephritis with albuminuria and casts. Cardiac and cerebral troubles may also occur. It has been suggested that the toxicity is due to the nitrate by conversion in the body into nitrite, but Dr. Lewin points out in the *Münchene Medizinische Wochenschrift* that the characteristic toxic action of nitrites is the formation of methæmoglobin, which, according to this author, cannot be obtained by the administration of bismuth subnitrate. He is of opinion, therefore, that the toxicity is entirely due to the bismuth. As a substitute for bismuth he proposes ferroso-ferric oxide (magnetic oxide of iron). It is not toxic and is impermeable to the *x*-rays to the same extent as bismuth. In fine powder it can be mixed with certain kinds of food, such as potato purée in the proportion of 30 per cent. or more. Experiments with animals have shown that it is especially useful in the study of intestinal peristalsis.

METASTASES in Gastric Carcinoma, as is well known, occur not rarely in situations where they may easily be missed unless systematically searched for, as, for example, in the supra-clavicular glands. A new site for early secondary deposits in the subjects of this disease is described in the *Albany Medical Annals* by Dr. Blumer, under the name of the rectal shelf. This is the vesico-rectal fold, wherein a metastatic growth can fairly often be found beyond the prostate in a certain proportion of cases, even when the primary disease is fairly early. The mass is described as feeling like a shelf projecting into the rectal cavity, carrying the rectal wall before it; the tumour is often of cartilaginous hardness, may or may not encircle the bowel as a primary rectal carcinoma does, but does not ulcerate the mucosa, and therefore does not cause the passage of blood or pus. Sometimes the mass is a little higher up, well beyond the prostate, and only just palpable by the examining finger. In dealing with gastric carcinoma the existence of metastases is a point which very materially affects the operative procedures and even the diagnosis itself; and if these observations are confirmed it will evidently become the duty of surgeons to examine the rectum as a routine before exploring the abdomen in a suspected case of gastric carcinoma.

---

THE exact effect of Ether and Chloroform upon the Kidneys and their functions is a matter which does not seem yet to be finally decided. Some physiologists believe that both drugs, but especially ether, cause temporary albuminuria and the passage of casts in a majority of patients. Others say that chloroform is the more liable to initiate albuminuria than is ether, but less likely to aggravate existing lesions and to set up suppression or hæmaturia. It has also been noticed that in delayed chloroform poisoning the kidneys show fatty degeneration. Again, operations on the kidneys are often attended by considerable shock, for which reason ether has been advocated against chloroform; and the constrained lateral position, in which respiration must necessarily be a little interfered with, has been adduced in the same sense. In cystoscopy when the ureteric orifices are to be examined it has been said that chloroform is preferable, as it does not check the secretion of urine in the way ether does. Some of these points have been reinvestigated by Dr. J. W. Bovée, who read a paper recently before the American Gynæcological Society. He finds that both ether and chloroform reduce the secretion of urine, and that this effect is greater in the case of chloroform; under the latter the proportion of urea remains normal, whereas under ether it is diminished. Also that neither anæsthetic has any constant or appreciable effect in producing casts or albuminuria.

---

THE Value of Percussion as an aid to the Diagnosis of Fractures of the Skull is highly esteemed by Mr. J. H. Pringle, who narrates in the *Edinburgh Medical Journal* three cases in which he was able to localise by this means intra-cranial hæmorrhage under a fracture. In examining the skull in this way it is advisable to make sure that the mouth is either shut or open throughout the percussion, as otherwise slight modifications may be introduced; it is also necessary to remember that any considerable œdema or hæmatoma must be discounted. The notes elicited by immediate percussion with the finger, preferably, but not necessarily, on the shaved scalp, are compared: (1) along the sagittal suture of the two sides, (2) over the parietal and occipital bones, and (3) very especially over each temporal fossa. When a fracture exists in the neighbourhood of these areas, the note is often lowered in pitch over the fracture zone, and in addition to this a definite crackpot quality is introduced in many cases. The latter is particularly to be observed when there is comminution, but also not uncommonly over the salient of an angular fracture. For either sign it is probably necessary that a fracture or fissure should extend a certain distance over the surface of the skull, and in fractures which are purely basal the alteration of the percussion note is not to be found at all. Some patients, in whom no alteration in the percussion note could be obtained over a suspected fracture, complained of pain always elicited in one place or one area. Mr. Pringle is inclined to regard this as an indication of fracture because in other patients the same complaint was produced, after recovery of consciousness, on percussing exactly those places where dulness or cracked pot sounds had been obtained whilst insensible. This sign, however, he regards as of less value than the other.

---

THE Abdominal Reflex as a diagnostic and also as a prognostic sign in Typhoid Fever was studied in the first instance by Dr. J. D. Rolleston. The main conclusions that he drew from his observations were as follows: That the abdominal reflex is affected in a very large number of cases of enteric fever, the percentage of cases in which it is entirely lost exceeding those in which its normal activity is only diminished. From its absence under the age of fifty being confined to certain nervous diseases and acute abdominal conditions, notably appendicitis and enteric fever, the absence of the abdominal reflex in a given case of continued pyrexia in any patient below fifty is of considerable value. The comparatively transient nature of the affection of the abdominal reflex in enteric fever is a striking contrast to the more chronic affection of the knee and ankle-joints in diseases associated with peripheral neuritis, *e.g.* diphtheria. Return of a lost reflex, and, *a fortiori*, resumption of its normal activity, are valuable indications of commencing convalescence, and often correspond with lysis and characteristic changes in the fæces and urine. The objective sign of return of the reflex is often associated with the return of the subjective feeling of ticklishness normal to the individual. In reappearance of pyrexia in convalescence, the condition of the abdominal reflex is a valuable index of the nature of the pyrexia. That is to say, its disappearance or its becoming sluggish would point to a relapse. No constant relation exists between the condition of the abdominal reflex and that of the tendon reflexes.

At a recent meeting of the Société Médicale des Hôpitaux, Lermoyez and Aubertin drew attention to the dangers which may result from unrestricted use of Adrenaline, a drug which is so often used by patients in various nasal sprays, douches, etc., without any supervision by the medical attendant. The author's experiments were carried out on rabbits. A number of these animals were subjected to a long course of nasal sprayings. In no case were the authors able to find subsequent atheroma of the vessels; but in some of the animals distinct evidences were found of cardiac hypertrophy and hyperplasia of the suprarenal bodies. In one case death resulted from acute œdema of the lung. Although these results are sketchy and inconclusive, they may attract further attention to the need for caution in the indiscriminate and unrestricted prescribing of potent animal sera and extracts; and to the desirability of constant clinical and laboratory research into the side-issues and remote possibilities of serum-therapy and organo-therapy.

Fabre and Roubier, in the *Lyon Médicale*, publish a study of Syphilitic Sciatica. In the author's opinion this condition is most frequently met with as a tertiary phenomenon, but may occur and be overlooked in the secondary stage. When occurring in the latter stage it is most frequently partial, and limited to a segment of the nerve, *e.g.* to that which supplies the buttock. The course is capricious, and unaccountable remissions and exacerbations, with nocturnal paroxysms of pain, are common. The condition quickly yields to antispecific treatment. When occurring in the tertiary stage it presents the ordinary symptoms of sciatica, with intense persistent pain along the whole course of the nerve. There has recently been a tendency to recognise two types of sciatica, namely, that of the nerve-trunk proper, and that of the nerve roots. Diagnosis between these two forms is by no means easy. Some authorities lay stress on the pain caused in the nerve by coughing and sneezing, which they regard as proof that the trouble is in the nerve roots. This sign is, however, not pathognomonic. Much more trustworthy is the presence of bands of hyperæsthesia or anæsthesia following the distribution of the affected nerve roots. In order of frequency the nerves affected are the fifth lumbar, first sacral, second sacral, fourth sacral, and, lastly, the third sacral.

The results of the first year's work of the special ophthalmic ward in St. Paul's Hospital, Liverpool, are summarised in the *Ophthalmoscope* by Mr. Nimmo Walker, to whom the conception and initiation of the scheme are due. The foundation from which everything is built up is the enforcement of notification of ophthalmia neonatorum by the Health Authority of the City, which suspends or reports to the Central Midwives Board those who neglect this duty. By this means early information is obtained in the great majority of cases, and whenever possible both mother and child are admitted as in-patients to the special ward for the purpose, unless the parents can afford to employ a private practitioner. After eighteen months' trial Mr. Walker commits himself to several definite statements. Experience shows that the theoretical objection of possible injury to the mothers by being moved so soon after parturition is not borne out in practice. Next the superiority of this in-patient treatment over out-patient methods is proved by results. Thirdly, the duration of disease varies directly, and the chance of complete recovery inversely with the length of time between the onset of the disease and the admission to hospital. The tabulated statement of the cases proves how valuable this work is, and the municipal authorities of Liverpool are to be congratulated upon the success of their co-operation with a charitable institution.

The following remarkable case illustrates the fact that dentists should invariably be most careful in the Disinfection of their Instuments, preferably by boiling. It also illustrates the unexpected ways in which a chancre may be caused. The full description will be found in the *Bulletin du Syndicat des Chirurgiens Dentistes.* The gist of the case is as follows: A medical man was consulted by a youth 12 years of age, who complained of a swelling of his nose. The first impression was that the lesion was an abscess, but later it turned out to be an undoubted hard chancre of the naso-labial sulcus with surrounding induration. Upon careful inquiry it was ascertained that the lad was a mechanician apprenticed to a dentist. He had, in common, apparently, with many other mechanics, acquired the deplorable habit of finishing off the polish on his instruments by rubbing them upon the side of his nose. By an unlucky chance one of the dentist's patients was actively syphilitic. Simple contact with the patient's buccal mucosa and secretions when the secondary eruption was fully out had infected the tools, and one of these had in turn led to transmission of the infection to the unlucky lad.

The results of some experiments by Van Bogaert with Serum-therapy in the treatment of Nephritis are published in *La Province Médicale.* The serum used was that of Teissier, which is derived from the renal vein of the goat. In all, nine cases were treated by subcutaneous injection. The first case was that of an acute nephritis with uræmic cerebral symptoms and eclampsia. The effect of the injections was the rapid amelioration of all symptoms caused by the renal insufficiency, though the scarlatinal symptoms, such as otitis, enlargement of the glands, arthritis, etc., were scarcely influenced. Equally good results were obtained in the second case, one of chronic nephritis in a tuberculous subject; the patient before the serum was at the onset of uræmia, and is now in a condition of renal sufficiency, the nephritis, however, persisting. The third case, in which the symptoms were mainly of cardiac origin, died of heart failure on the eighth day. In the next two cases the nephritis was respectively gouty and scarlatinal, and marked benefit followed the injections. The remaining trials were for the most part unsuccessful, but they were upon cases of extreme gravity.

